# A New Paradigm for Pandemic Preparedness

**DOI:** 10.1007/s40471-023-00336-w

**Published:** 2023-11-09

**Authors:** Nina H. Fefferman, John S. McAlister, Belinda S. Akpa, Kelechi Akwataghibe, Fahim Tasneema Azad, Katherine Barkley, Amanda Bleichrodt, Michael J. Blum, L. Bourouiba, Yana Bromberg, K. Selçuk Candan, Gerardo Chowell, Erin Clancey, Fawn A. Cothran, Sharon N. DeWitte, Pilar Fernandez, David Finnoff, D. T. Flaherty, Nathaniel L. Gibson, Natalie Harris, Qiang He, Eric T. Lofgren, Debra L. Miller, James Moody, Kaitlin Muccio, Charles L. Nunn, Monica Papeș, Ioannis Ch. Paschalidis, Dana K. Pasquale, J. Michael Reed, Matthew B. Rogers, Courtney L. Schreiner, Elizabeth B. Strand, Clifford S. Swanson, Heather L. Szabo-Rogers, Sadie J. Ryan

**Affiliations:** 1 Department of Ecology and Evolutionary Biology, University of Tennessee, Knoxville, TN 37996-3140, USA; 2 University of Tennessee, National Institute for Mathematical and Biological Synthesis, Knoxville, TN, USA; 3 Department of Mathematics, University of Tennessee, Knoxville, TN, USA; 4 Department of Chemical & Biomolecular Engineering, University of Tennessee, Knoxville, TN, USA; 5 School of Computing and Augmented Intelligence (SCAI), Arizona State University, Tempe, AZ, USA; 6 Department of Economics, University of Wyoming, Laramie, WY, USA; 7 Georgia State University, Prior Second Century Initiative (2CI) Clusters, Atlanta, GA, USA; 8 Massachusetts Institute of Technology, Cambridge, MA, USA; 9 Department of Biology, Emory University, Atlanta, GA, USA; 10 Department of Computer Science, Emory University, Atlanta, GA, USA; 11 Department of Population Health Sciences, Georgia State University School of Public Health, Atlanta, GA, USA; 12 Paul G. Allen School for Global Health, Washington State University, Pullman, WA, USA; 13 National Alliance for Caregiving, Washington, DC, USA; 14 Institute of Behavioural Science and Department of Anthropology, University of Colorado, Boulder, CO, USA; 15 Department of Civil and Environmental Engineering, The University of Tennessee, Knoxville, TN, USA; 16 The University of Tennessee, Institute for a Secure and Sustainable Environment, Knoxville, TN, USA; 17 One Health Initiative, University of Tennessee, Knoxville, TN, USA; 18 Department of Sociology, Duke University, Durham, NC, USA; 19 Department of Biology, Tufts University, Medford, MA, USA; 20 Evolutionary Anthropology, Duke University, Durham, NC, USA; 21 Duke University, Duke Global Health Institute, Durham, NC, USA; 22 Triangle Center for Evolutionary Medicine, Duke University, Durham, NC, USA; 23 Department of Electrical and Computer Engineering, Boston University, Boston, MA, USA; 24 Duke Department of Population Health Sciences, Duke University School of Medicine, Durham, NC, USA; 25 Duke Network Analysis Center, Duke University, Durham, NC, USA; 26 Vaccine and Infectious Disease Organization (VIDO), University of Saskatchewan, Saskatoon, SK, Canada; 27 Colleges of Veterinary Medicine, University of Tennessee, Knoxville, TN, USA; 28 Social Work Center for Veterinary Social Work, University of Tennessee, Knoxville, TN, USA; 29 Department of Anatomy, Physiology and Pharmacology College of Medicine, University of Saskatchewan, Saskatoon, SK, Canada; 30 Department of Geography, Quantitative Disease Ecology and Conservation (QDEC) Lab, University of Florida, Gainesville, FL, USA; 31 University of Florida, Emerging Pathogens Institute, Gainesville, FL, USA; 32 College of Life Sciences, University of KwaZulu-Natal, Durban, South Africa

**Keywords:** Pandemic preparedness, Multidisciplinary science, Transdisciplinary science, Pandemic science

## Abstract

**Purpose of Review:**

Preparing for pandemics requires a degree of interdisciplinary work that is challenging under the current paradigm. This review summarizes the challenges faced by the field of pandemic science and proposes how to address them.

**Recent Findings:**

The structure of current siloed systems of research organizations hinders effective interdisciplinary pandemic research. Moreover, effective pandemic preparedness requires stakeholders in public policy and health to interact and integrate new findings rapidly, relying on a robust, responsive, and productive research domain. Neither of these requirements are well supported under the current system.

**Summary:**

We propose a new paradigm for pandemic preparedness wherein interdisciplinary research and close collaboration with public policy and health practitioners can improve our ability to prevent, detect, and treat pandemics through tighter integration among domains, rapid and accurate integration, and translation of science to public policy, outreach and education, and improved venues and incentives for sustainable and robust interdisciplinary work.

## Introduction

Despite the increasing threat, pandemics are remarkably hard to predict [1, 2], even with recent technological advances in the areas of detection, prevention, and treatment. Predicting pandemics is also difficult due to the wide range of factors that mediate, promote, or suppress the manifestation of infectious diseases. Contributing factors vary widely, ranging from biological characteristics of a pathogen or host immunology, to ecological drivers of a vector, and to sociodemographic constraints on the hosts [3], studied by a collection of sparsely connected disciplines. Infrastructural and policy barriers, such as a lack of funding, political will, streamlined public health communication, and public education about the scientific method, notions of certainty or lack of it, and the inherently dynamic nature of scientific evidence also hinder success. These gaps have contributed to an increase in public mistrust in science and public health recommendations and decreased adoption of health protective behaviors. The suite of disciplines that are all crucial to pandemic preparedness [4, 5], in both the scientific research and the public health and policy domains, is rarely truly interconnected.

To date, much of pandemic research involves examining the most recent pandemic to inform and guide responses to unfolding events. This phenomenological practice, although very important for developing, testing, and improving methods, provides limited capacity to predict pandemics or prepare for pandemics with unanticipated or different attributes (e.g., [6]). Because so many independent components can contribute to the development of pandemics, research targeted at understanding how previous pandemics arose can only provide a partial picture of the conditions that may lead to the next pandemic.

Research on pandemics also has a long history of simplification for solvability (e.g., [7, 8]). By design, distilling phenomena into component parts and connections is a simplification necessary to identify what to measure or model. Simplification can be necessary for the study of complex phenomena but nonetheless limits the use of knowledge and models for prediction, preparation, and response to future pandemics. Qualitative results from simple models can yield important insights about potential pandemic outcomes but give us little ability to *predict* and *prevent* the next pandemic with available information [9, 10]. Similarly, it is challenging to extend more highly specified models to predict future pandemics in part because different elements like inception and spread can be qualitatively and categorically different from past conditions, or a stochastic event might not be repeated.

Pandemic prediction and preparation are also constrained by the confederated structure of the science enterprise, made of a collection of siloed disciplines. While separation can be essential for advancing knowledge, siloed areas impose barriers to investigation of complex phenomena like pandemics [11, 12]. Consider two disciplinarians studying pandemics from two separate disciplines: the language of their disciplines, their networks of resources, and the audiences for their results may be entirely different. Thus, the separation of disciplines, however necessary it may be, limits our ability to derive knowledge and insights critical to advancing pandemic preparedness and response. For example, in the context of early pandemic identification and response, siloed communication of a number of entities, including human health clinicians, the community, and exotic animal veterinarians, and their associated diagnostic laboratories is thought to have significantly delayed recognition of the 1999 West Nile threat in the USA [13]. In the broader and more recent context of public health policy and response to emerging pandemics such as the Coronavirus disease-2019 (COVID-19), the gaps in understanding of the transmission of viruses through the air, rooted in the structural and historical separation of fields, slowed recognition of airborne transmission and impeded clear messaging on how best to slow COVID-19 transmission. In fact, revisiting the historical foundation of airborne transmission as defined in public health guidelines (1800s–present) at the time of COVID-19 onset illustrates the pendulum swing of scientific history. This demonstrated the urgent need for sustained infrastructure development for collaboration, synthesis of multiple disciplines, and the critical need for ongoing and streamlined integration of scientific results into public health practice for building a more resilient society with more sound, farsighted, and effective public health policies [14, 15].

Akin to the complications that can arise in the research domain, the separation of disciplines can also limit public health responses. For example, theoretical models projecting the course of a pandemic may suggest interventions that are not practical given resource-constraints and/or are potentially culturally inappropriate or even illegal. Furthermore, the same public health practitioners that are implementing public health responses may be unaware of the processes and capabilities of the other disciplines. For example, when working with jails, prisons, and other detention centers, a number of interventions that are straightforward to articulate—responsive releases, jail diversion, or the widespread reduction in how incarcerated individuals mix—can meet both political and legal resistance that make implementing them nonviable [16]. Similarly, when universities had to confront the COVID-19 pandemic’s impact on student populations, the considerations extended beyond strictly minimizing disease morbidity or mortality. Decisions included consideration of the quality of education, impact on the local economy, pressure from politicians, as well as alumni, donors, and parents’ groups (all potentially influenced by misinformation), and, ultimately, the university’s bottom line. Political will ultimately prove to be a significant deciding factor in the fate of many attempts to control the pandemic, on local, state, national, and global scales.

Just as critically, decades of underfunded public health have left us with a badly strained workforce, limited in their ability to meet these challenges. COVID-19 demonstrated that local public health forces, acting only as a stopgap until national level resources can be mobilized, are inadequate for addressing the emergency response needs of an entire nation without coordinated guidance. This leaves critical health jurisdictions—those serving rural populations, predominantly minoritized populations, economically disadvantaged areas, etc.—under a tremendous amount of strain. Similarly, the “Boom and Bust” cycle of pandemic research and public health funding in response to public health emergencies—the “Boom” of which occurs at best only mid-emergency—makes recruiting and retaining a skilled workforce at all levels of pandemic research and public health difficult. For example, as an increasing number of universities consider introducing public health into their offerings at both the graduate and undergraduate level, there is a very real question in the wake of COVID-19 as to how viable public health is as a career path, and whether the realities of public health training and compensation can meet our needs for the future. On the research side, the same boom and bust research funding cycle makes it impossible to sustain deep and high-risk/high-reward, transformative research on grand challenges, let alone those requiring the hard initial work of profound integration of disparate disciplines. Even when emergency resources are made available on an ad hoc basis for one disciplinarian, it may not be readily available to others. A lack of sustained infrastructure means many may remain unaware that the resources exist or lack the language necessary to properly seek them out. Similarly, building the necessary connections between disciplines required to answer the actual challenge takes sustained support and effort. Recognizing and breaking down barriers across scientific domains and the structural issues of sustainable funding for pandemic research and public health are key steps towards advancing truly impactful pandemic preparedness.

The profit-based incentives for some public-health entities (including hospitals, which become profit necessities to public health practices when they cannot operate with the funding they already have available) mean that the current structure incentivizes compromising preparation in favor of short-term profits. Even if, over a long period of time, preparing for is less costly than responding to a crisis, this preparation requires redundancy and stockpiling, which are seen as economic inefficiencies [17, 18]. This tradeoff between resilience of the entire system (i.e., society) in the long term and efficiency of sub-parts of the system (e.g., hospitals) in the short term means that a number of public health stakeholders are not able to afford adequate preparation.

We propose that many of the barriers both from the public health and research perspectives can be overcome by increasing efforts to focus on interdisciplinary work. Bridging gaps between disciplines will allow researchers to leverage powerful tools from related disciplines and answer questions which do not fall neatly into a particular subfield. It is not that interdisciplinary study has been ignored or underestimated, but rather there are systematic structural barriers to effective and sustained interdisciplinary research that need to be addressed to substantially improve our ability to prepare for pandemics. On an individual level, treating interdisciplinary work as a group of disciplinarians doing work alongside one another prevents proper training of true interdisciplinarians. On a systemic level, incentive structures and funding cycles reinforce the ineffective paradigm of disciplinarian tool-building versus application and new insights specific to a disease system or application. By improved training for interdisciplinarians and systems which incentivize longitudinal collaborative work across both the research and public health domain, we can work towards a new paradigm of pandemic preparedness. With improved capacity to answer questions which span many disciplines and communicate findings across disciplines more effectively, we can improve surveillance, prevention, detection, and control of pandemics.

### Questions in Pandemic Preparedness

#### In the Research Division

Recalcitrant barriers to interdisciplinary research can impede progress on crucial questions about pandemic preparedness. Addressing questions about pandemic emergence and behavior would provide practical guidance for data collection regarding a pathogen, substrate, or system and for making predictions about associated pandemic outcomes. For example, being able to classify contact networks on which pathogen(s) might spread before a pathogen is introduced would allow for more accurate and precise response or more effective community design as a preventative measure (e.g., [19, 20]). Similarly, understanding which genomic sequences result in pathogenicity would improve our ability to specify surveillance of known pathogens of concern and potential etiological agents [21, 22].

Similar questions can focus on the factors that determine pandemic emergence. It would be helpful to be able to measure a system under non-pandemic conditions and make predictions about how a pandemic might emerge from that system [23]. For instance, we might ask how to predict which contacts in a network persist and which new contacts emerge during an outbreak (e.g., [24]) by studying the network under non-pandemic conditions [25]. We might also ask what selective pressures enhance or suppress mutations for new pathogenicity [26], or we might want to know how climate change or other changes in ecological conditions might alter a pathogen’s realized niche [27••] and if such an ecological shift could result in a pandemic [28–30]. Crucially, none of these factors alone cause or prevent outbreaks; a greater understanding of how many of these factors interact to promote or inhibit one another vastly improves our ability to detect and respond to pandemics. It might also improve our ability to prevent pandemics by highlighting what aspects of the systems that govern global populations are apt to promote the emergence of new pathogens and enhancing the resilience of those systems [31, 32••].

Yet another class of questions are those regarding how to classify systems and system behaviors into meaningful and actionable designations. By focusing on abstraction of the characteristics, we might predict system behavior without having to describe each component in complete detail. For example, suppose we knew that, in the social domain, when perceived severity is in the intensity interval a_1_-a_2_, then people will stay home and reduce their interactions with others by a proportion that falls in the interval b_1_-b_2_. This would allow us to make control decisions quickly without having to know the precise relationship between risk perception and response. Likewise, suppose we could classify viruses by their number of distinct mammalian hosts and by their probability to jump between species. If this classification could be mapped to a probability of introduction to humans with a parameter for frequency of exposure, we could then target surveillance efforts or prepare for those pathogens that pose the greatest threat to human populations. We might also seek to classify probabilities of horizontal transmission of a factor like antibiotic resistance between pathogens by the overlap of their host ranges. Such a classification would allow for more accurate predictions about how climate change, and the resulting shifts in host ranges, might affect the likelihood that a pathogen could lead to a pandemic. One of the largest barriers to classifying systems in this way is the inherent high dimensionality of the systems involved. The siloed research and funding gaps that limit addressing multifactorial, interdisciplinary questions (e.g., how genomic information links to phenotype and function of most pathogens, and by association, how to link evolution and transmission from the point of view of a pathogen with the actual environmental conditions it experiences and that shape its adaptation). Each of the preceding classifications fall into separate fields, but they are all impacted by factors from outside those fields. The most effective classification of systems by pandemic risk would require a great deal of interdisciplinary work starting from problems like these and understanding their interactions.

The final class of questions addresses the outcomes of possible actions to intervene in, or mitigate the progression of, a threat. Mathematical models from control and decision theory have provided elegant tools for identifying optimal mechanisms for intervention and discovering their optimal timing of application to achieve desired objectives (though only if the scenario can be well approximated by a system of mathematical equations simple enough to satisfy the assumptions and limitations of the analytic methods available). Extensions of these techniques to address constraints in which mechanisms can be affected, or by how much; ensuring that transient dynamics do not dip below allowable thresholds; allowing prior performance of attempted intervention to shape future constraints; and extension over spatial, temporal, and sociopolitical landscapes of impact have also been foci of recent efforts. Of course, identifying an optimal (or even locally maximal) path to control is also predicated on defining the objective itself. In pandemic science, these objectives are as nuanced as the threats. Goals of control can be to minimize the numbers of infections, deaths, symptomatic people requiring care at any one time (e.g., flattening the curve), or long-term adverse outcomes. It could instead focus on maximizing adoption of protective behaviors, numbers of vectors eliminated, or number of patients with access to care. Rather than a straightforward minimization or maximization of a numerical objective, we can also have qualitative goals that make strict mathematical control theory more difficult to apply, leaving us in the obligately multidisciplinary realm at the intersection of many disciplines. For example, we may have a goal of egalitarian outcomes among groups. We may also face nuanced constraints, such as attempting to minimize damage to other aspects of health and social well-being, functioning economies, ongoing diplomatic cooperation among governments, and more.

All these questions are, by nature, interdisciplinary. Answers to these questions and others, as well as improved methods of practice would vastly increase our ability to prevent, detect, and treat pandemics, but progress hinges on interdisciplinary work. Even if each question above could be sorted nicely into a social, biological, or ecological domain, answering them cannot be accomplished within a single field. For example, classifying contact networks on which disease might spread is research that would find a home in epidemiological journals but requires collaboration between sociologists, virologists/microbiologists, and perhaps immunologists. Determining the selective pressures that might enhance or suppress pathogenic mutations is a question in evolutionary biology and functional genomics as well as biophysics, physiology, community ecology, and epidemiology. Examining relationships between host ranges, horizontal transmission, and pandemic risk of a particular pathogen can be thought of as an ecological pursuit but again requires a great many other disciplines from anthropology to microbiology to physics to fully characterize. Moreover, answers to these questions immediately pose new ones focusing on the health systems that overlap high-risk ecological areas. Furthermore, the answers to any of these questions are not actionable without public health and policy experts translating findings into practice.

#### In the Public Health Domain

There are challenges to the field of pandemic preparedness which come from outside the field. Relatively few scientists have formal training, experience, or resources about how to interface with policy makers. This includes how to identify and approach such policy makers, and how to effectively communicate findings that are relevant to them, for example, analyzing and accounting for policy making tension between precision of research findings and multifactorial and multi-stakeholder constraints shaping final decisions, when preparing for effective communication. Similarly, policy decisions are often made rapidly, sometimes relying on preprints, provisional results, and expert opinion. This is an unfamiliar, and often uncomfortable, timeline for many researchers who then must abandon focusing only results that would be suitable for peer-reviewed publication, the gold standard of quality-control in science. This creates challenges, especially for junior researchers, or researchers with small research groups, who find themselves caught between the need to publish and the desire to serve and meet the urgency of the moment and support policy makers.

Decades of underfunded public health have left many health jurisdictions without the expertise or bandwidth to prepare for and/or detect an incipient pandemic. The challenges range from simply knowing which researchers to call and what their capabilities are (and likewise, researchers knowing who to contact in agencies that often experience high turnover), to facilitating data sharing or leveraging expertise to help plan and implement interventions or forecast local cases. The types of relationships that allow for close partnerships with local health officials and the ability to use researchers’ resources, personnel, and expertise to supplement local public health response require years to build, with few or no resources to back them, especially in vulnerable communities with a swath of competing demands for both time and money.

Outside the formal lines of public health, other pressures have complicated how researchers respond to public health emergencies. Questions from the origins of the pandemic to routine childhood vaccination schedules have become politicized, leading to the insertion of political considerations into discussions of specific research programs. Alongside this, public trust in science has eroded, aided by political agendas, social media, and the dissemination of misinformation. This leads to an extremely complex information landscape for public health research—one for which few public health researchers are well prepared.

Adopting an integrated, interdisciplinary approach to address questions about pandemics from the onset stage of problem/question definition would constitute a substantive step towards advancing pandemic preparedness. Such an early interdisciplinary framework could enable the early integration of all stakeholder considerations (e.g., potential pandemic treaties, vaccine stockpiling regulation, testing policies, availability of hospital facilities, school closure policies, or vector control strategy) into the questions being defined and considered [3, 33–35]. It could also, for example, facilitate characterization of pandemic tipping points and thus provide a basis for identifying early warning signals of outbreaks. Individual communities could have models built from geographically relevant data to improve the timing and resolution of surveillance and prevention. More advanced interdisciplinary understanding of emergence could also strengthen capacity to implement more effective practices to prevent and ameliorate pandemics. Furthermore, having a more unified investigative approach that draws on a multitude of disciplines might help address some associated challenges, such as sustained investment in public health infrastructure, federally mandated sharing of data, and a research cloud to provide the infrastructure for computationally demanding work, akin to infrastructure and investment in weather monitoring and forecasting over the years.

### Challenges of Interdisciplinary Study

Realizing the promise of interdisciplinary work requires overcoming longstanding challenges and impracticalities. There are several classes of challenges to consider: (1) those relating to researchers and practitioners themselves, (2) those that are a product of the system that researchers and practitioners work within, and (3) those that relate to the external pressures that shape the system that supports work, including policies that shape funding landscapes.

The success of interdisciplinary work is not simply the result of gathering a diverse set of disciplinary thinkers together with a common goal. By definition, disciplinary training relies on shaping expectations about how to both describe and then interrogate a system. Groups of diverse disciplinarians have been trained into disparate languages, systems of thought, and methods for approaching their work. The contributions of each disciplinarian may be critical to the combined insight of collaborative work, but the collaboration itself is frequently aided by the inclusion of focused interdisciplinarians: people whose disciplinary depth of knowledge may not equal their colleagues (though nothing prevents that), but whose motivation and specialty lies specifically in translating among disciplines. (It should be noted that many interdisciplinarians themselves specialize in bridging the interface between/among specific disciplines, developing their own deep knowledge in those areas.) In designing teams to tackle truly complex challenges that span disciplines, it may be ideal to purposefully include a balanced mix of strictly disciplinary and specifically interdisciplinary voices.

Even when partners from multiple disciplines may be interested and committed to interdisciplinary work, finding research questions of mutual interest that are “impactful” in two or more fields can be challenging. This can stem from basic problems, such as fully understanding what the other field brings to the table (e.g., not viewing computer scientists as software developers) or working out a shared language between fields. But the issues can be more subtle as well. For example, real, actionable results may emerge in some fields with the application of relatively simple analytical techniques such as classification or clustering-based methods. These applied problems may produce impactful research in epidemiology or clinical practice and be of real and measurable benefit to human health but still present a conundrum for collaborators in computer science, where it is entirely possible that the application of well-studied and understood techniques such as decision trees and logistic regression does not constitute sufficiently innovative disciplinary work to be considered promotion-level or degree-granting scholarship. At the same time, the push for more sophisticated methods to address those needs may lessen the ultimate ability for the research to be applied and have an impact in the other domain.

Interdisciplinary work can be stymied by systemic challenges. For example, there may not be sufficiently coherent communication and evaluation to sustain integrative consideration of a problem [36, 37]. The big questions and goals in pandemic preparedness can be too large for a handful of teams to adequately answer. Because of this, many teams across many disciplines research different layers of the same question. Separate threads of research examining the same question can be disconnected and thus become reciprocally unhelpful towards achieving the original (common) goal. Even when interdisciplinary researchers have results, it can be hard to find places to publish, particularly if those results are not groundbreaking. “Smaller” results do the important job of bridging gaps between disciplines but nonetheless may be cast aside by journals for not advancing a particular disciplinary understanding. Similarly, demonstrating quality interdisciplinary work can be a challenge unto itself.

External pressures that shape the current system of investigative work can directly and indirectly impede interdisciplinary study. Policy actions themselves impact critical dynamics in real time. For example, funding cycles that produce incentivized bursts of interest in a particular disease can counterintuitively impede advancement of the field as a whole. Cycles of incentivized funding can reinforce the paradigm of studying the last pandemic relevant to the method at hand [38]. Although this approach can derive valuable knowledge and insight, it does little to further our ability to detect, prevent, or even treat the next pandemic which is almost certainly going to be qualitatively different from the last. There are key moments to fund multi-disciplinary teams to advance prediction and response capacity during the lulls between major pandemics. Funding that is always responsive, rather than prescriptive, will continue to struggle to harvest deep and truly innovative insights, nor can it enable effective adaptation, translation, and operationalization of knowledge gains over necessary timelines.

### Potential Interdisciplinary Solutions

Barriers to interdisciplinary study are not easily solved because they are not faults of a particular discipline or short-comings of a particular research method. Rather, barriers are often necessary consequences of the current research ecosystem. Solutions, therefore, must be forged as changes to the ecosystem as well as external pressures shaping the ecosystem.

The past 10 years have involved increased discussion in the community about how to train researchers to undertake inter- and multidisciplinary studies. Many programs have focused on cross-training, where disciplinarians in one area spend time immersed (via research rotation or coursework) in another area. Others have implemented collaborative, team-based approaches that construct cohorts from various disciplines and encourage purposeful discussion of the challenges in communication and collaboration that arise among them. These efforts are all worthy in their own ways, but also cannot be relied upon as solutions to meet the challenges of the research community. Focusing on the next generation of researchers as “native interdisciplinarians” means that there may be critical mismatches between interests, competencies, and expectations between junior researchers and their mentors in ways that may undercut the success of the endeavor.

We propose that any solutions that focus on building novel intrinsic interdisciplinary capacity may also come by modifying the basis of existing incentive structures. Much of the current incentive structure relies on traditional metrics such as the number of papers published as well as the venue, which can reinforce disciplinary silos and prevent interdisciplinary collaboration. For example, when a paper is published in a journal curated for a particular discipline, the results may not come to the attention of researchers active in other fields and public health professionals who could benefit from the work—or may not carry value for promotion or other practical concerns. Valuing other metrics of productivity and creating more demand for cross discipline and cross-domain work (i.e., the communication of traditionally disciplinary work to those outside of the discipline or original area of impact) as a way to complement the current incentive structure may generate more research to address interdisciplinary questions in pandemic preparedness. Shifting the basis of the current incentive structure could also lead to greater emphasis on critically important, yet non-traditional, outcomes, like rapid knowledge gain from quick course research on an emerging disease. Incentives should be put in place to expand capacity and willingness to quickly pivot to work towards providing rapid results that may not be suitable for conventional outlets like peer-review journals. Doing so could incentivize careers of research in ready service towards answering key questions, including those that might arise during the early stages of a pandemic.

Building a readily available reference list of high-profile publications dedicated to sharing research across disciplines and domains could help researchers and practitioners learn different vocabularies and languages, which might incentivize a convergence to a shared language and pool of resources across the entire field. Elevated exposure might also increase access to knowledge and resources from complementary disciplines. Such high-profile publications might serve as a clearinghouse for work that informs professional practices. It also incentivizes researchers to investigate interdisciplinary questions, which could positively feedback to strengthen reputational and professional gains that might come from publishing in a dedicated venue. While deeper or narrower studies are still of great value, the nature of academia is that broader, higher profile work can shape how a community coalesces around a question and foment more collaborative methods to address challenges. Of course, such a resource will require careful infrastructural research and design to ensure that it adds utility to the community, rather than simply providing one more equivalently confusing resource in an already crowded field.

New incentive structures and high-profile venues for interdisciplinary publications about pandemic preparedness may fail in the absence of opportunities for researchers and public health professionals to collaborate. Mechanisms that promote collaboration, like institutes and centers where individuals from different communities can come together to pursue work of shared interest, can be crucial to the advancement of interdisciplinary study. Such centers and institutes can act as a convening place, bringing people from different disciplines together, and providing them with sufficient resources to commence new collaborations. Establishing institutes for pandemic preparedness and centers for collaboration across pandemic-related disciplines could foster progress towards a more unified and therefore productive field. The recently launched US National Science Foundation efforts to establish Predictive Intelligence for Pandemic Prevention Institutes purposefully addresses these goals, but the purposeful focus of these centers should not be restricted to the foci inherent in the NSF’s remit. Such centers will be the strongest in incentivizing change if they can complement each other, providing leadership in how to construct a functioning network of expertise, beginning with basic research but then also extend through implementation, involving all the agencies and foundations that touch on the scientific, medical, health, sociological, economic, infrastructural, technological, and political aspects of pandemic preparedness. Incentivizing career choices to lead centers and institutes could further advance opportunities for interdisciplinary study. Directors often act as liaisons, capable of connecting oftentimes disparate ideas and people across disciplines and divisions. Serving in this capacity can, however, come at a cost because it does not necessarily yield outputs that satisfy the current incentive structures. Incentivizing individuals to engage a wide breadth of scholarship and large networks (i.e., over depth) could further elevate capacity to identify new areas of growth and help identify new ways to work cohesively towards solving critical questions in pandemic preparedness.

Greater infrastructure for outreach and engagement could also help advance the field. Dedicated outreach initiatives would help connect researchers and public health professionals with the aim of facilitating the development of collaborations. Such initiatives could also serve to encourage researchers and practitioners to consider interdisciplinary pursuits by raising awareness of tools, methods, and processes of investigation. Similarly, providing community portals for models might encourage the development and adoption of common experimental model systems as well as the means for testing and validating models. Increasing accessibility to the broader community would make model comparison across disciplines much easier and help lay a foundation for a common language across the field of pandemic preparedness. An online repository could provide unrestricted access to information on published work from pandemic-related fields, data sets that are available for study, and analytical tools, which could further help draw together researchers and practitioners across disciplines and divisions. Such efforts have been very successfully implemented at the intersection of some disciplines, for example, consider *Physionet* [39], a dedicated, curated, and maintained database of physiological data, aimed at stimulating current research and new investigations of complex biomedical and physiologic signals. Over the years, it has served as a gold-standard benchmark for models and methodologies that can compare their performance or predictive power on a common identical extensive curated and accepted dataset. On a broader scale, weather data and modeling, e.g., *NOAA* [40] or *NCAR* [41] databases and models, show the societal and economic benefits of national level, empowering of integration of innovation and resources for development of surveillance, research, and policy to ensure national capacity in weather system research and its operationalization via forecasting, forever changing the economic, agricultural, and emergency responses involving weather. This historic success of weather forecasting was achieved despite initial resistance to the very idea of the possibility of weather forecasting, prior to chaos theory and computing, and despite the multifaceted, interdisciplinary chaotic complex and multiscale weather and climate system. Indeed, such a system, like the one shaping pandemics, does involve integration of a range of disparate disciplines, from mathematics, chemistry, and radiative and fluid physics to biology to cite a few, while also requiring overcoming grand technological challenges from devices and surveillance to disparate data curation and management, to tremendously costly multiscale computation and data integration, even if we assume a solution to the data availability challenges themselves. It is hard to believe that once upon a time, weather forecasting was considered impossible, and in fact not supported, and considered a major cost with little benefits (e.g., [42, 43]).

Interdisciplinary work does more than just allow for more informed, impactful, and efficient research, it may also afford solutions to parallel challenges in public health practices as well as broader forces that shape the underpinnings of pandemic preparedness (e.g., [44]). As we potentially enter the “Pandemicene” [27••], the research approaches and funding patterns for pandemic preparedness must be treated as ongoing necessary public good, rather than one-off emergencies, to be dealt with strictly with a bolus of funding midway through the crisis. As has been the case with weather forecasting, once centralized and supported with permanent infrastructure, steady, predictable funding for pandemic preparedness would allow for strengthening and coordination of public health surveillance and data sharing infrastructure, as well as the sustained training of public health workforce, fostering relationships between public health agencies on the state and local level and researchers, and improving the evidence base upon which our pandemic response plans are built. Surges of funding might still be needed—as with weather related disasters—to address singular threats, but the scale of these surges could potentially be much smaller, while the return on the long-term public good investments be much greater. This would then enable sustained and innovative systems of resilience aimed at preventing radical measures, such as generalized economic and education shutdowns, and thus also enable tremendous economic and life gains. Similarly, our approach to pandemic preparedness must explore new ways to harness the energy and enthusiasm of researchers. Frameworks and spaces beyond the next paper or conference proceeding that allows cross-disciplinary learning and longer term research to be viewed as public good and so valuable to the public, the researchers, and the institutions within which they reside must be fostered. This potentially spans everything from recognizing that supporting policy makers involves a cost in both time and resources that may not then cleanly manifest as traditional scientific publications, to coming to an understanding of the communal value that education and engagement by a researcher with the public provides for all. This engagement may require tailored, platform-specific effort, since it will likely require a diverse range of media to be effective, particularly in time of misinformation, increasing societal disconnection, and rampant loss of confidence in the political, scientific, and public health enterprises as agents of good for all. Innovation must also extend to science education in the public sphere. An understanding must be fostered as to what science does and does not do. Beyond cross-disciplinary work to improve the state of pandemic science, cross-disciplinary work is needed to improve how we disseminate that science. Experts in science education and science communication must be at the forefront of these activities, integrated into cross-disciplinary teams to inform pandemic preparedness. Beyond merely suggesting approaches at the outset, their expertise is needed in adapting to a changing environment, as well as defining what the hallmarks of a successful communication program look like. This includes considering novel sources of public education about infectious diseases—including sources that exist outside a pandemic. Popular media has used everything from zombie fiction [45] to games such as Plague Inc. and World of Warcraft [46] to allow people to discuss contagious disease threats—and public health responses to them—without the politicization and stigma of real diseases. These are, at least, interesting one-off case studies, but perhaps, this approach could be considered more broadly in the future, to allow public health education to meet the public where they are.

More broadly, art about pandemics, risk management, and history of pandemics and scientific discovery in general provokes new ways for a non-scientific audience to critically consider what role they themselves play in pandemic preparedness (e.g., [47]). Moving beyond the use of art for education and outreach to communicate results of scientific discovery (e.g. [48–54]), art allows a personal and social contextualization of challenging problems (e.g., [55, 56]). During the COVID-19, an essay in the journal Public Health detailed a wide diversity of perspectives shown through the art of children in India ranging from fear for a struggling world to responsibility to one another [57]. Perhaps, artwork ranging from music to television and movies can be an important tool for demonstrating a diversity of perspectives on pandemic risk or even a helpful way to introduce crucial ideas in pandemic preparedness to an audience that wouldn’t otherwise consider them.

We must also consider how to restore the public’s battered trust in science and public health. How do we prevent public health messaging from becoming politicized? How do we best communicate uncertainty, and science’s need to revise its messaging in the face of new evidence? There are clear analogies here to weather forecasting, which has considerably more expertise in communicating uncertain predictions to the public. And how does public health respond to those outside the field—and within it—who are willing to artificially downplay real uncertainty to advance their goals? Furthermore, the role of the tech giants that control many of the social media platforms is important, implementing mechanisms to control and refute misinformation spread over these media. Whether it is desirable or not, pandemic preparedness finds itself in a complex social and political environment, and experts in those areas must have a seat at the table.

### Vision for the Future

**Fig. 1:**
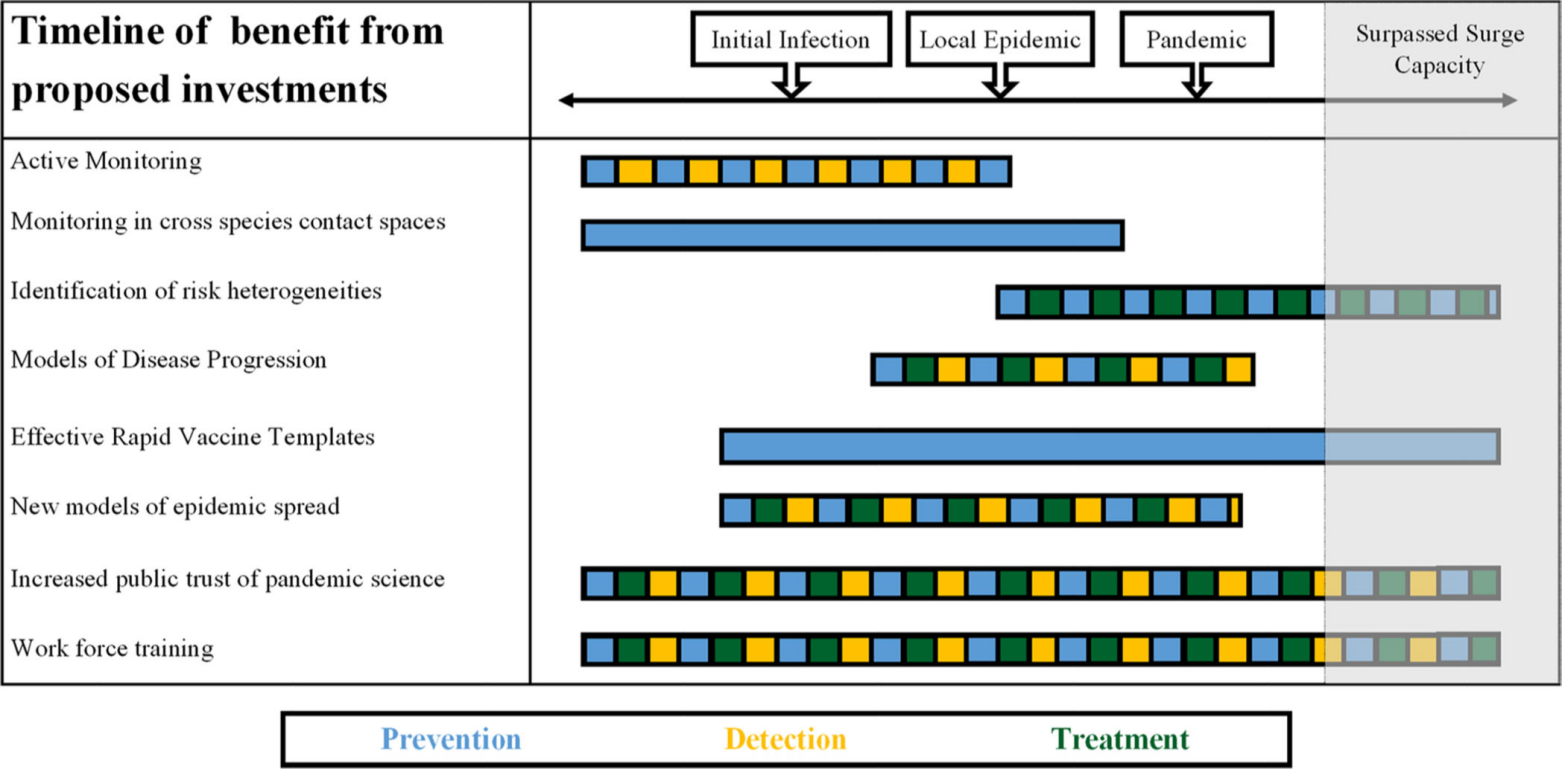
A timeline demonstrating when we would observe a benefit for each proposed investment. Before the start of the pandemic (to the left) represents the prevention and detection phase and after the pandemic (to the right) represents the treatment and control phase. Each bar is colored to demonstrate which dimensions of pandemic preparedness are impacted directly

It is complicated to imagine a world in which siloed traditions of the scientific disciplines are truly broken down, especially without obstructing key training in those domains. Instead, holistic interdisciplinary umbrella groups, such as centers of excellence, synthesis centers and networks, and key spaces to interface with non-governmental organizations (NGOs), governmental agencies, and independent think tanks, should be made available and indeed involve sequestering dedicated and committed time and staffing. The spaces in which science can address complex systems such as pandemics will need specific remits to exist [58, 59] (Fig. 1). Specifically, agency staff do not normally have the bandwidth to join workshops during business hours and cannot always participate in resulting publications, public reports, or even make recommendations for actions or investments. This is not unique to the USA, nor to country-level decision makers.

The future we envision for the field of pandemic preparedness departs from the current constellation of isolated researchers and public health professionals. We argue that the field ought to shift to foster innovation through interdisciplinary study of past, current, and future pandemics. Some lessons can be learned from efforts to reshape the field of disaster preparedness and efforts to address chronic public health concerns. Consider, for example, the broad actions that have been organized to advance treatment of cancer that build beyond distinct efforts to address specific forms of cancer (e.g., Koch Institute for Integrative Cancer Research [60] and similar others). Likewise with heart disease, responses involve broader actions to respond to many potential causes of impairment (e.g., Comprehensive Heart Attack Center Certifications [61]). Pandemic preparedness should involve like-minded efforts—in other words, just as we prepare for disasters, we need also to prepare for pandemics. The recommendations we present will better enable researchers and public health professionals to contribute to broader campaigns to prepare for the next pandemic.

Different questions can be posed and answered more readily under a shifted pandemic preparedness paradigm. An emphasis on interdisciplinary study would allow for continued learning about a particular pandemic or a particular type of pandemic, while also promoting work towards answering more general questions that require more cohesive investigation. For instance, greater emphasis might direct attention towards understanding the rarity of pandemics. Work might be directed towards understanding whether pandemics are rare only because the small set of things that lead to them are independently rare, or because the particular confluences of independently common things are itself rare. The current paradigm provides little opportunity to answer these and similar questions. Consequently, we remain poorly prepared to prevent or face future pandemics.

A compilation of small and large changes is required to modify our collective approach to pandemic preparedness. Implementing some or all the proposed changes will take time and consideration, but we believe that from them will emerge great improvements in the way we can prepare for an ever-increasing threat. Through increased investment in interdisciplinary training and solutions, with common sustained infrastructure, researchers, and practitioners in the field of pandemic preparedness, can improve the way we prevent, detect, and control pandemics.
